# Genome-wide association study for ashy stem blight resistance in USDA common bean germplasm

**DOI:** 10.3389/fpls.2025.1590571

**Published:** 2025-05-21

**Authors:** Ainong Shi, Diego M. Viteri, Angela M. Linares-Ramírez, Haizheng Xiong, Senyu Chen

**Affiliations:** ^1^ Department of Horticulture, University of Arkansas, Fayetteville, AR, United States; ^2^ Department of Agro-Environmental Sciences, University of Puerto Rico, Isabela Research Substation, Isabela, Puerto Rico; ^3^ Department of Agro-Environmental Sciences, University of Puerto Rico, Lajas Research Substation, Lajas, Puerto Rico; ^4^ Southern Research & Outreach Center, University of Minnesota, Waseca, MN, United States

**Keywords:** ashy stem blight, common bean, *Macrophomina phaseolina*, *Phaseolus vulgaris*, quantitative trait loci, genome-wide association study

## Abstract

Ashy stem blight (ASB), caused by the fungus *Macrophomina phaseolina* (Tassi) Goidanich, poses a significant threat to common bean (*Phaseolus vulgaris* L.) cultivation worldwide. Due to the limited effectiveness of fungicides against ASB, the development of genetically resistant cultivars offers an eco-friendly and sustainable management strategy. This study aimed to accelerate genetic improvement in common bean by integrating molecular breeding tools with conventional breeding approaches to enhance ASB resistance. A total of 335 common bean germplasm accessions from the United States Department of Agriculture (USDA) Germplasm Resources Information Network (GRIN) collection were evaluated for their response to the PRI21 *M. phaseolina* isolate. A genome-wide association study (GWAS) was conducted using 87,193 high-quality single-nucleotide polymorphisms (SNPs) and four statistical models—Bayesian-information and Linkage-disequilibrium Iteratively Nested Keyway (BLINK), mixed linear model (MLM), multiple-locus MLM (MLMM), and general linear model (GLM)—implemented in GAPIT3. Twenty-three accessions had intermediate resistance, with Andean PI 173208 and PI 264786 showing the lowest disease severity scores (<3.7) to PRI21. Four quantitative trait loci (QTLs) were identified on chromosomes *Pv*02, *Pv*04, *Pv*05, and *Pv*10 across all accessions. Additionally, three QTLs were detected on *Pv*01, *Pv*02, and *Pv*11 in Mesoamerican accessions, while three QTLs were found on *Pv*02, *Pv*04, and *Pv*06 in Andean accessions. The two candidate genes *Phvul.002G046300* [leucine-rich repeat (LRR) family protein] and *Phvul.002G046500* (receptor-like protein kinase 1) were identified on chromosome *Pv*02 as being associated with ASB resistance. These SNP markers linked to these QTLs may be valuable for marker-assisted selection in common bean breeding programs aimed at improving ASB resistance.

## Introduction

Ashy stem blight (ASB) is a significant disease affecting common bean (*Phaseolus vulgaris* L.) in the United States and worldwide (Ambachew et al., 2021; Kaur et al., 2012). It is caused by the fungus *Macrophomina phaseolina* (Tassi) Goidanich, which can infect plant tissue throughout the entire growing season (Islam et al., 2012; Kaur et al., 2012; Viteri and Linares, 2022). Yield losses can reach values over 60% in susceptible common bean cultivars (Mayek et al., 2003; Viteri and Linares, 2022). The most characteristic symptom is the stem blight at vegetative and reproductive stages in susceptible *Phaseolus* spp. genotypes (Kaur et al., 2012; Vázquez et al., 2024; Viteri and Miranda, 2024). The primary infection structure, microsclerotia, can persist in the soil for over a decade (Kaur et al., 2012; Short et al., 1980). Additionally, the fungus is seed-transmitted, and variations in aggressiveness among isolates have been reported (Mayek et al., 2001a; Miklas et al., 1998a; Vázquez et al., 2024; Viteri and Linares, 2017).

Resistance to ASB has been reported in common bean (*P. vulgaris*), scarlet runner bean (*Phaseolus coccineus* L.), and tepary bean (*Phaseolus acutifolius* A. Gray). For instance, several common bean genotypes, including A 195, BAT 477, IPA 1, Negro Tacaná, Negro Perla, ‘PC 50’, PRA154, PRA155, ‘San Cristobal 83’, TARS-MST1, XAN 176, and recently developed breeding lines UPR-Mp-22, UPR-Mp-34, UPR-Mp-37, UPR-Mp-42, and UPR-Mp-48, have shown resistance to ASB in multiple environments (Mayek et al., 2001b; Miklas et al., 1998b; Pastor-Corrales and Abawi, 1988; Porch et al., 2012; Viteri and Linares, 2017, 2022a; Vázquez et al., 2024; Viteri et al., 2019, 2023; 2024b). Additionally, tepary bean accessions Mex-114, PI 440806, and PI 321637 demonstrated resistance in field evaluations (Miklas et al., 1998a). However, higher levels of resistance were observed in scarlet runner bean accession PI 183412 in ASB screenings conducted in the greenhouse (Viteri and Miranda, 2024).

Genetic studies have identified that the *Mp-1* and *Mp-2* complementary dominant genes derived from BAT 477 conferred resistance to ASB (Olaya et al., 1996). Mayek et al. (2009) reported two dominant genes with double recessive epistasis and nine quantitative trait loci (QTLs) associated with field resistance to ASB and were introgressed from BAT 477. Likewise, nine QTLs on chromosomes *Pv*03, *Pv*05, *Pv*06, *Pv*08, *Pv*09, and *Pv*10 were linked to ASB resistance and were also derived from BAT 477 (Méndez et al., 2017). Further, five QTLs on chromosomes *Pv*04, *Pv*06, *Pv*07, and *Pv*08 provided field resistance to ASB and were derived from black common bean XAN 176 (Miklas et al., 1998b). More recently, Viteri and Linares (2019) identified two recessive genes in ‘PC 50’/‘Othello’ and ‘Badillo’/PR1144-5, while one dominant gene conferred resistance to ASB in the A 195/‘PC 50’ population. Viteri et al. (2022) reported a novel QTL on *Pv*03 chromosome for ASB resistance in a BAT 477/NY6020–4 cross, along with two single-nucleotide polymorphisms (SNPs) and a candidate gene. Additionally, Viteri et al. (2024a) identified a QTL on *Pv*07 chromosome for ASB resistance derived from PRA154 Andean common bean.

SNPs, due to their abundance, cost-effectiveness, and compatibility with high-throughput genotyping, have become invaluable tools in genome-wide mapping, association studies, diversity analysis, and gene tagging in plant breeding (Collard and Mackill, 2008; Xu and Crouch, 2008). Identifying SNP markers enables breeders to enhance selection for biotic and abiotic stress resistance, expediting the development of elite cultivars with improved stress tolerance or resistance to diseases in common bean breeding programs. The declining cost of genotyping and advances in statistical methods have further strengthened the potential of genome-wide association studies (GWASs) for improving complex traits in crops. GWAS, which utilizes diverse populations and high-density SNP markers, has successfully identified causal genes associated with a broad range of agronomic traits (Li et al., 2013; Morris et al., 2013; Yano et al., 2016). As molecular breeding continues to evolve, integrating GWAS, marker-assisted selection (MAS), and genomic selection (GS) into breeding programs will be crucial for accelerating genetic gains and enhancing crop resilience.

The United States Department of Agriculture (USDA) Germplasm Resources Information Network (GRIN) houses over 10,000 common bean accessions, with phenotypic data available for more than 30 traits. For instance, GWASs have been conducted in common bean to identify QTLs or candidate genes for anthracnose [caused by the fungus *Colletotrichum lindemuthianum* (Sacc. & Magn.)] (Zuiderveen et al., 2016); bacterial wilt [caused by *Curtobacterium flaccumfaciens* pv. *flaccumfaciens* (Hedges) Collins & Jones] (Zia et al., 2022); bacterial brown spot [caused by *Pseudomonas syringae* pv. *syringae* van Hall], common bacterial blight (caused by *Xanthomonas axonopodis* pv. *phaseoli* and *Xanthomonas fuscans* subsp. *fuscans* Smith), and halo bacterial blight [caused by *P. syringae* pv. *phaseolicola* Burkholder) (Soler-Garzón et al., 2024); *Fusarium* wilt [caused by *Fusarium oxysporum* Schlecht. f. sp. *phaseoli* Kendrick & Snyder] (Paulino et al., 2021); and soybean cyst nematode [caused by *Heterodera glycines* Ichinohe (Tylenchida: Heteroderidae)] (Shi et al., 2021), among other pathogens. However, a GWAS to identify SNP markers linked with QTLs and their candidate genes for ASB resistance has not yet been evaluated in USDA common bean accessions. The primary goal of this research was to enhance and accelerate common bean genetic improvement through molecular breeding, complementing ongoing classical breeding efforts to expedite genetic gain and develop improved cultivars with ASB resistance. Specifically, this study aimed to evaluate USDA GRIN common bean germplasm accessions for ASB resistance and conduct a GWAS to identify SNP markers associated with ASB resistance in common bean.

## Materials and methods

### Plant materials

This study utilized 335 common bean germplasm accessions from the USDA GRIN collection. These accessions were originally collected from 45 countries, with the majority (74%, 248 accessions) originating from 10 primary contributing countries. The largest contributors included Mexico (60 accessions), China (39), Bulgaria (37), the United States (31), Turkey (19), India (16), North Macedonia (14), Hungary (12), France (11), and the Netherlands (9) (**Supplementary Table S1**). Throughout this article, supplementary tables and figures will be denoted by “S” (e.g., **Supplementary Table S1**, **Supplementary Figure S1**).

### Phenotyping of ashy stem blight resistance

‘Othello’ was used as the susceptible control (Viteri and Linares, 2017, 2022a), whereas the Andean breeding line PRA154 was used as the partial resistance control to ASB (Viteri et al., 2024a) in this study. Due to space limitations, two separate experiments were conducted. In October 2022, 105 common bean genotypes were evaluated in a greenhouse at the Lajas Research Substation at the University of Puerto Rico. Additionally, 230 accessions, along with both parents, were screened in a greenhouse at the Isabela Research Substation in March 2023. The temperatures in both greenhouses ranged from 29°C to 38°C, with a relative humidity between 40% and 60% at the time of inoculation, providing optimal conditions for ASB development (Pastor-Corrales and Abawi, 1988; Vázquez et al., 2024; Viteri and Linares, 2022a).

A completely randomized block design with three replications was employed for both experiments. Two seeds planted in a 16-cm-diameter plastic plot containing Pro-Mix BX at pH 5.9 per replication were used in each greenhouse. Each pot was fertilized with 200 mL of 20-20–20 NutriLeaf^®^ (Miller Chemical & Fertilizer, Hanover, PA, USA) at the V2 growth stage. Inoculation was performed once with the PRI21 *M. phaseolina* isolate (Viteri et al., 2023) at the fourth internode on 3-week-old plants (~V4 growth stage) using the cut-stem method (Viteri and Linares, 2017). Disease severity was assessed using a 1–9 scale at 42 days post-inoculation.

The 1–9 scale was defined as follows:

- **1**: No signs of pathogen infection;
- **3**: Fungal growth restricted to the first node above or below the point of inoculation;
- **6**: *M. phaseolina* reached the second node above or below the inoculation site; and
- **9**: The pathogen passed the third node below the point of inoculation, potentially causing plant death (Viteri and Linares, 2017).

Plants with scores of 1–3 were classified as resistant, 4–6 as intermediate, and 7–9 as susceptible (Viteri and Linares, 2017, 2022a).

### Phenotypic data analysis

Disease severity index (DSI) was used for data analysis, where DSI = 100* disease score of the accession in each replicate/highest score 9. DSI of ASB phenotypic data was analyzed by analysis of variance (ANOVA) using the general linear model procedure of JMP Genomics 9 (SAS Institute, 2012). The descriptive statistics were generated using “Tabulate”, and the distribution of the data was drawn using “Distribution” of JMP Genomics 9 (SAS Institute, 2012). The least-squares mean to ASB isolate PRI21 resistance for each accession from ANOVA was used as the phenotypic data for GWAS.

The broad-sense heritability (H^2^) was estimated, using the following formula (Holland et al., 2003).


$$H^{2}=100*\sigma_{G}^{2}/[\sigma_{G}^{2}+\sigma_{E}^{2}]$$

where σ^2G^ is the total genetic variance, σ^2E^ is the residual variance, and r is the number of replications. The estimates for σ^2G^ and σ^2E^ are σ^2E^ = MS_E_ and σ^2G^ = (MS_G_ − MS_E_)/r.

### Genotyping

DNA was extracted from fresh leaves of bean plants using the cetyltrimethylammonium bromide (CTBA) method. The genomic DNA was then randomly sheared into short fragments of approximately 350 bp each. Library construction was performed using the NEBNext^®^ DNA Library Prep Kit, following the instructions provided by Novogene (http://en.novogene.com/). This process included end repair, dA-tailing, and ligation with NEBNext adapters, followed by PCR enrichment using P5 and indexed P7 oligos to select fragments sized between 300 and 500 bp. After purification and quality assessment using a Qubit^®^ 2.0 fluorometer to determine the library concentration and the Agilent^®^ 2100 Bioanalyzer to assess insert size, quantitative real-time PCR (qPCR) was performed to evaluate the effective concentration of each library. Libraries with appropriate insert sizes and effective concentrations exceeding 2 nM were considered qualified for Illumina^®^ high-throughput sequencing. Qualified DNA libraries were pooled based on their effective concentrations and expected data production, and pair-end sequencing was performed on the Illumina^®^ sequencing platform, generating PE150 bp reads. The common bean genome reference Pvulgaris 442_v2.1 from the Phytozome website (https://genome.jgi.doe.gov/portal/pages/dynamicOrganismDownload.jsf?organism=Pvulgaris) served as the reference for mapping the short reads using the Burrows-Wheeler Aligner (BWA; 0.7.8-r455). SAMtools (0.1.19-44428cd) was used for sorting the resulting Binary Alignment Map (BAM) files and removing duplicate reads, while Picard (v.1.111) was employed to merge BAM files from the same sample.

SNP and InDel detection and filtering were carried out using GATK software (v.3.5), with annotation performed using ANNOVAR. A total of 24.4 million SNPs were identified across 11 chromosomes in the 335 accessions, ranging from 1.47 million SNPs on chromosome 6 to 2.9 million SNPs on chromosome 8. After applying filtering criteria, including a minor allele frequency >2%, missing allele <10%, and heterogeneity rate <30%, 0.7 million high-quality SNPs were retained. For subsequent analyses, 87,193 high-quality SNPs, randomly selected from approximately 10,000 SNPs per chromosome, were used (**Supplementary Figure S1**; https://doi.org/10.6084/m9.figshare.28464020.v1).

### Principal component analysis and genetic diversity

In this study, 87,193 SNPs were included in the principal component analysis (PCA) and genetic diversity analysis. PCA and genetic diversity were analyzed using GAPIT3 (Wang and Zhang, 2021), with PCA components set from 2 to 10 and neighbor-joining (NJ) tree settings from 2 to 10. Phylogenetic trees were constructed using the NJ method in GAPIT3. Genetic diversity was assessed for all 335 accessions, as well as for two sub-populations (Q1 and Q2), using GAPIT3. Additionally, genetic diversity for the ASB-resistant accessions was evaluated using MEGA 7 (Kumar et al., 2016). Phylogenetic trees for these accessions were constructed based on the maximum likelihood method, with parameters described by Shi et al. (2021, 2022).

### Association analysis

Genome-wide association studies were conducted using various models, including Bayesian-information and Linkage-disequilibrium Iteratively Nested Keyway (BLINK), mixed linear model (MLM), multiple-locus MLM (MLMM), and general linear model (GLM), in GAPIT3 (Wang and Zhang, 2021). The analysis was performed on a panel of 335 accessions using 87,193 SNPs. Multiple models were employed to identify robust and consistent SNP markers associated with resistance to ASB in common bean.

The significance threshold for associations was determined using Bonferroni correction of *p*-values with an α = 0.05 (0.05/number of SNPs). A logarithm of odds (LOD) value of 6.25 [here, LOD is used instead of −log(*p*-value)] was employed as the significance threshold based on the 87,193 SNPs. Additionally, associated SNPs were selected with LOD >4.0 or 3.5 for different association panels (Q1 and Q2) in this study. Furthermore, a *t*-test was conducted for all 87,176 SNPs in the panel of 335 accessions using Visual Basic code in Microsoft Excel 2020, and *t*-test results for significant SNPs were obtained using GAPIT3.

### Candidate gene prediction

Candidate genes associated with ASB resistance were identified within a 50-kb region on both sides of the significant SNPs (Shi et al., 2021; Zhang et al., 2016). The candidate genes were extracted from the reference annotation of the common bean genome, specifically from the Pvulgaris 442_v2.1 assembly, available on the Phytozome website at https://genome.jgi.doe.gov/portal/pages/dynamicOrganismDownload.jsf?organism=Pvulgaris.

## Results

### ASB resistance evaluation

The susceptible control ‘Othello’ exhibited high susceptibility, with a mean score of 9 on the disease severity scale (1–9), indicating significant infection following inoculation with the PRI21 *M. phaseolina* isolate. The resistant control, PRA154, showed an intermediate response, with disease severity scores between 3.7 and 5.5, as expected in both locations. This suggested that common bean genotypes with intermediate resistance to ASB were present in the study.

**Figure 1:**
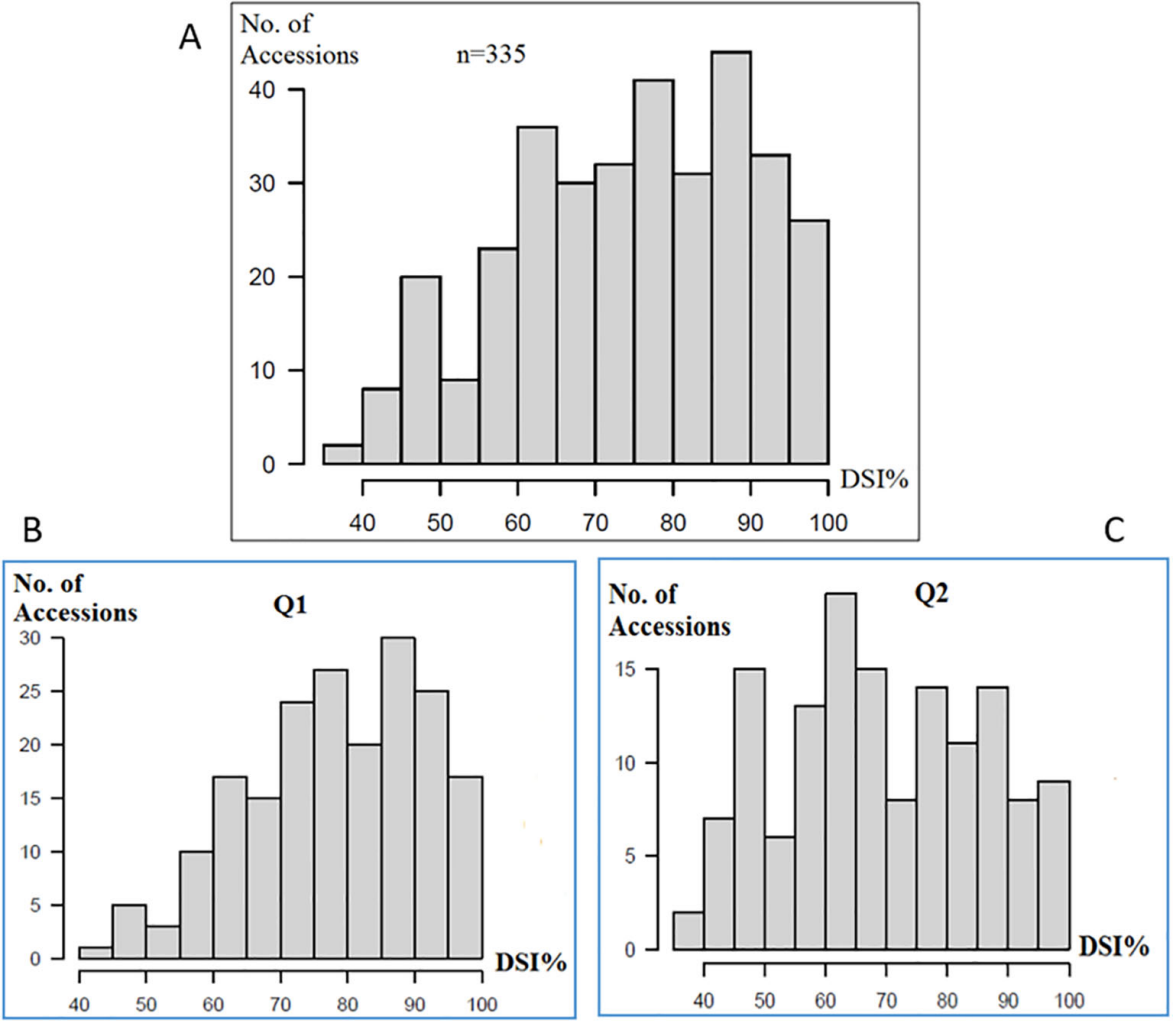
Distribution of ashy stem blight isolate PRI21 disease severity index (DSI) on **(A)** 335 USDA common bean accessions, **(B)** Q1 of 194 accessions of Mesoamerican origin, and **(C)** Q2 of 141 accessions of Andean domestication, where x-axis presents DSI% and y-axis presents number of accessions. DSI, disease severity index.

Significant differences (*p* ≤ 0.0001) were observed among the genotypes for the DSI (**Supplementary Table S2**, **Figure 1A**). The DSI distribution for the PRI21 *M. phaseolina* isolate across 335 USDA common bean accessions showed a near-normal distribution, slightly skewed toward the susceptible end, with more susceptible accessions. Both the Mesoamerican (Q1, 194 accessions) and Andean (Q2, 141 accessions) sub-populations displayed a similar distribution (**Figure 1**), indicating that ASB resistance in these panels is controlled by multiple genes.

The DSI values for *M. phaseolina* isolate PRI21 ranged from 37.0 (PI 264786 from France) to 100 (W6 12376, W6 16677, and PI 361206), with an average of 74.4, standard deviation (SD) of 15.14, standard error (SE) of 0.83, and coefficient of variation (CV) of 20.35% (**Supplementary Table S1C**). The disease score distribution (1–9) followed a similar trend, with a range from 3.3 to 9, averaging 6.7, SD of 1.36, SE of 0.07, and CV of 20.35% (**Supplementary Table S1C**). Among the 335 accessions, 23 exhibited moderate resistance to ASB, with disease scores below 4.5 and DSI values less than 50% (**Table 1**).

**Table 1:** List of 23 intermediate-resistant common bean accessions, with ID, name, origin (country), and their ashy stem blight disease score and disease severity index (DSI), ordered by two groups, followed by origin (country).

Accession_ID	Accession	Country	2-Cluster	ASB_PRI21.DSI	ASB_PRI21.Score
PI430590_China_Q1	PI 430590	China	Q1	44.4	4.0
PI309834_Costa Rica_Q1	PI 309834	Costa Rica	Q1	46.3	4.2
PI 150405_El Salvador_Q1	PI150405	El Salvador	Q1	46.3	4.2
PI310692_Guatemala_Q1	PI 310692	Guatemala	Q1	46.3	4.2
PI 209473_Mexico_Q1	PI 209473	Mexico	Q1	48.1	4.3
PI 313749_Mexico_Q1	PI 313749	Mexico	Q1	48.1	4.3
PI 638885_Argentina_Q2	PI 638885	Argentina	Q2	44.4	4.0
PI 173208_Australia_Q2	PI 173208	Australia	Q2	38.9	3.5
PI161952_Belgium_Q2	PI 161952	Belgium	Q2	40.7	3.7
W6 12201_Bulgaria_Q2	W6 12201	Bulgaria	Q2	48.1	4.3
W612235_Bulgaria_Q2	W6 12235	Bulgaria	Q2	46.3	4.2
PI368771_Serbia&Montenegro_Q2	PI 368771	Former Serbia and Montenegro	Q2	46.3	4.2
PI264786_France_Q2	PI 264786	France	Q2	37.0	3.3
PI264142_Germany_Q2	PI 264142	Germany	Q2	46.3	4.2
PI414817_Hungary_Q2	PI 414817	Hungary	Q2	48.1	4.3
PI163116_India_Q2	PI 163116	India	Q2	40.7	3.7
PI226523_Iran_Q2	PI 226523	Iran	Q2	44.4	4.0
PI 309702_Mexico_Q2	PI 309702	Mexico	Q2	42.6	3.8
PI293355_Peru_Q2	PI 293355	Peru	Q2	42.6	3.8
PI 262163_Switzerland_Q2	PI 262163	Switzerland	Q2	40.7	3.7
PI169790_Turkey_Q2	PI 169790	Turkey	Q2	48.1	4.3
PI169880_Turkey_Q2	PI 169880	Turkey	Q2	46.3	4.2
PI339414_Turkey_Q2	PI 339414	Turkey	Q2	48.1	4.3

The ANOVA revealed that the broad-sense heritability for resistance to *M. phaseolina* isolate PRI21 was estimated to be 64.3% (**Supplementary Table S2**), suggesting that ASB resistance is highly heritable.

### Genetic diversity and population structure analysis

**Figure 2:**
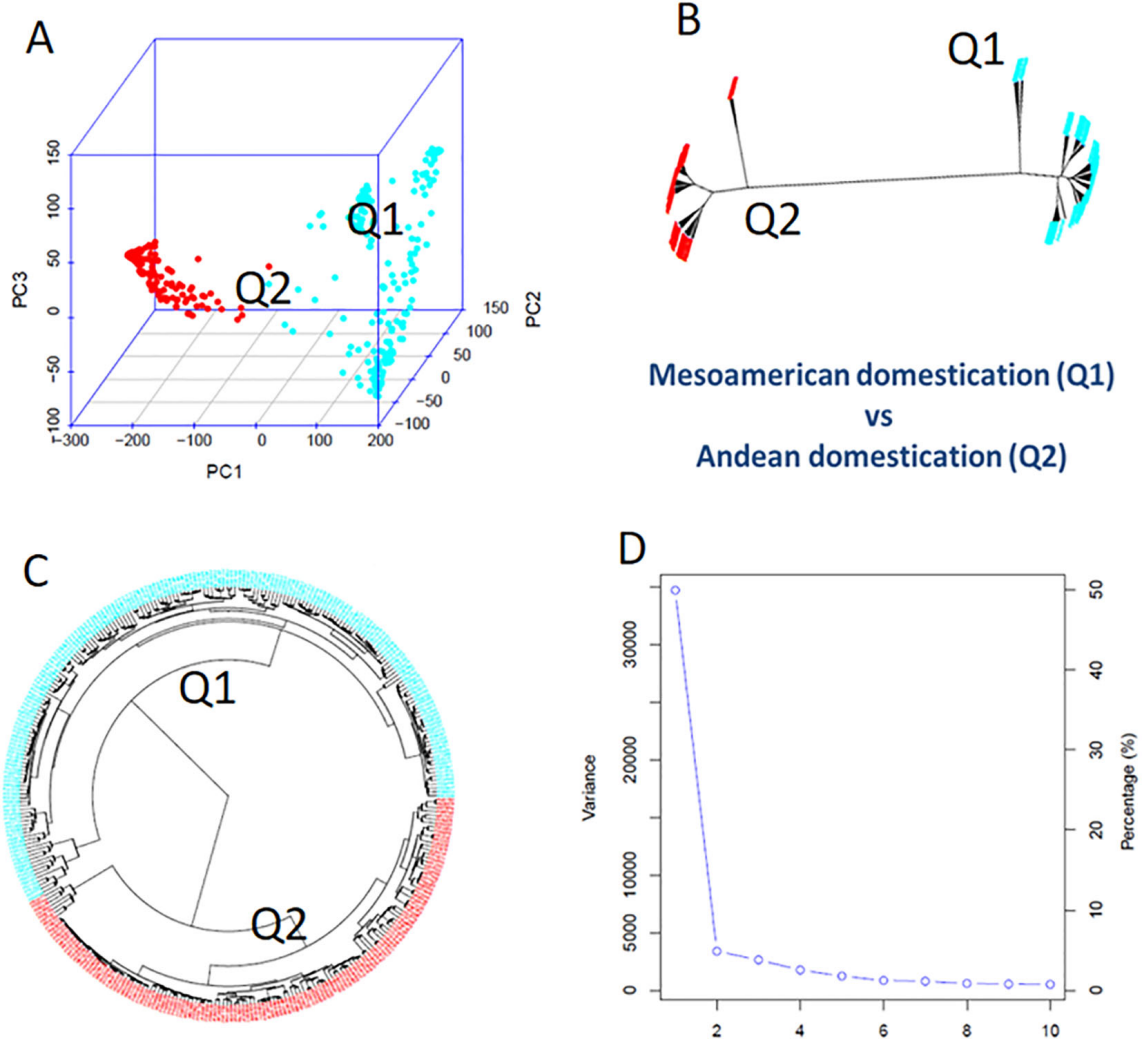
Population genetic diversity analysis in the association panel consisted of 335 USDA common bean germplasm accessions. **(A)** 3D graphical plot of the principal component analysis (PCA). **(B, C)** Phylogenetic trees (**B**, unrooted; **C**, fan) drawn by neighbor-joining (NJ) method in two sub-populations. **(D)** PCA eigenvalue plot drawn by GAPIT3.

Using GAPIT3, the 335 common bean accessions were divided into two distinct clusters (sub-populations), labeled Q1 and Q2 (**Figure 2**). This division was based on several analyses: 1) a 3D graphical plot of the principal component analysis (PCA) (**Figure 2A**), 2) phylogenetic trees constructed using the NJ method (**Figure 2B**, no-root, and **Figure 2C**, ring), and 3) a PCA eigenvalue plot (**Figure 2D**). Additionally, the kinship plot confirmed the presence of these two groups among the 335 accessions (**Supplementary Figure S2**). Each of the 335 accessions was assigned to one of the two clusters (Q1 or Q2) (**Supplementary Table S1A**), and the resulting Q-matrix with two clusters was applied to the GWAS analysis.

The Q1 sub-population, consisting of 194 accessions, was further subdivided into three clusters based on PCA and phylogenetic analysis (PCA components ranging from 2 to 10 in GAPIT3) (**Supplementary Figure S3**). Similarly, the Q2 sub-population, comprising 141 accessions, was divided into two clusters (**Supplementary Figure S4**).

### Genome-wide association study

In this study, GWAS for resistance to *M. phaseolina* isolate PRI21 was conducted using four models in GAPIT3—BLINK, MLMM, MLM, and GLM—along with a *t*-test for each SNP. The GWAS analysis was performed across three common bean panels: the full set (335 accessions), Q1 (194 accessions), and Q2 (141 accessions).

**Figure 3:**
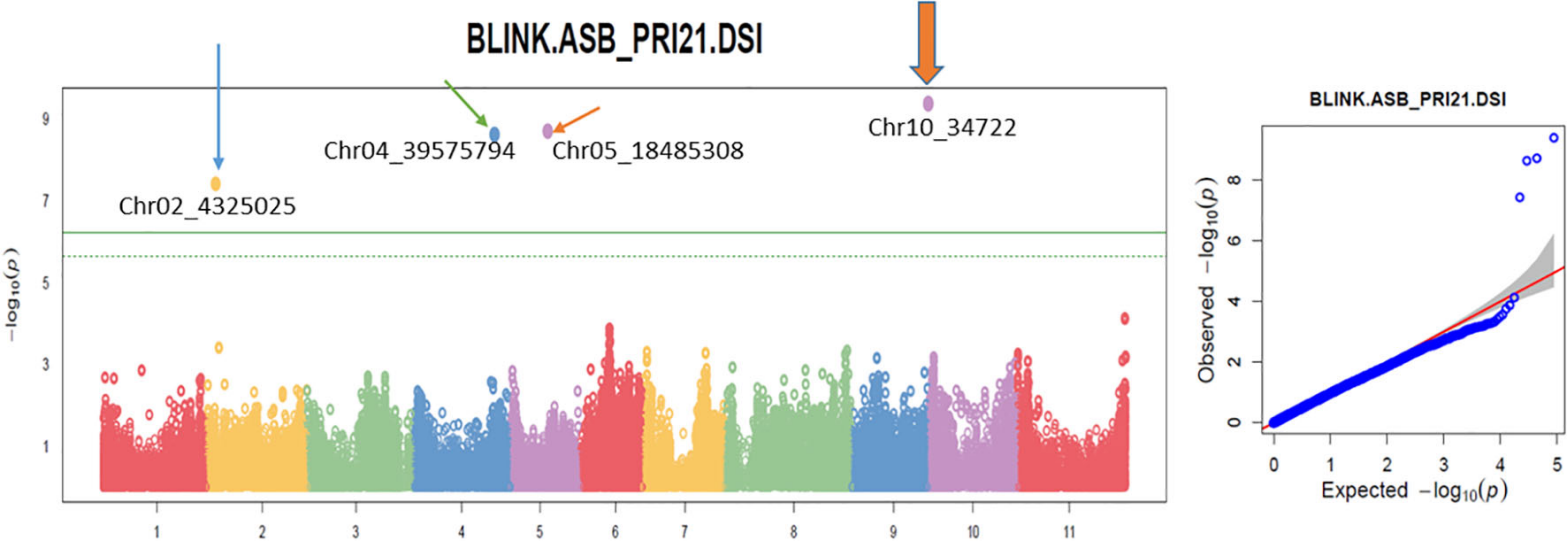
Multiple Manhattan plot (left) and QQ plot (right) in BLINK model in GAPIT3 for ashy stem blight isolate PRI21 disease severity index (DSI) on an association panel consisting of 335 USDA common bean accessions. The Manhattan plot (left) illustrates common bean 11 chromosomes on the x-axis and LOD [−log(*p*-value)] values on the y-axis. The QQ plot (right) displays LOD [−log(*p*-value)] values on the x-axis and expected LOD [−log(*p*-value)] values on the y-axis. BLINK, Bayesian-information and Linkage-disequilibrium Iteratively Nested Keyway; LOD, logarithm of odds.

**Figure 4:**
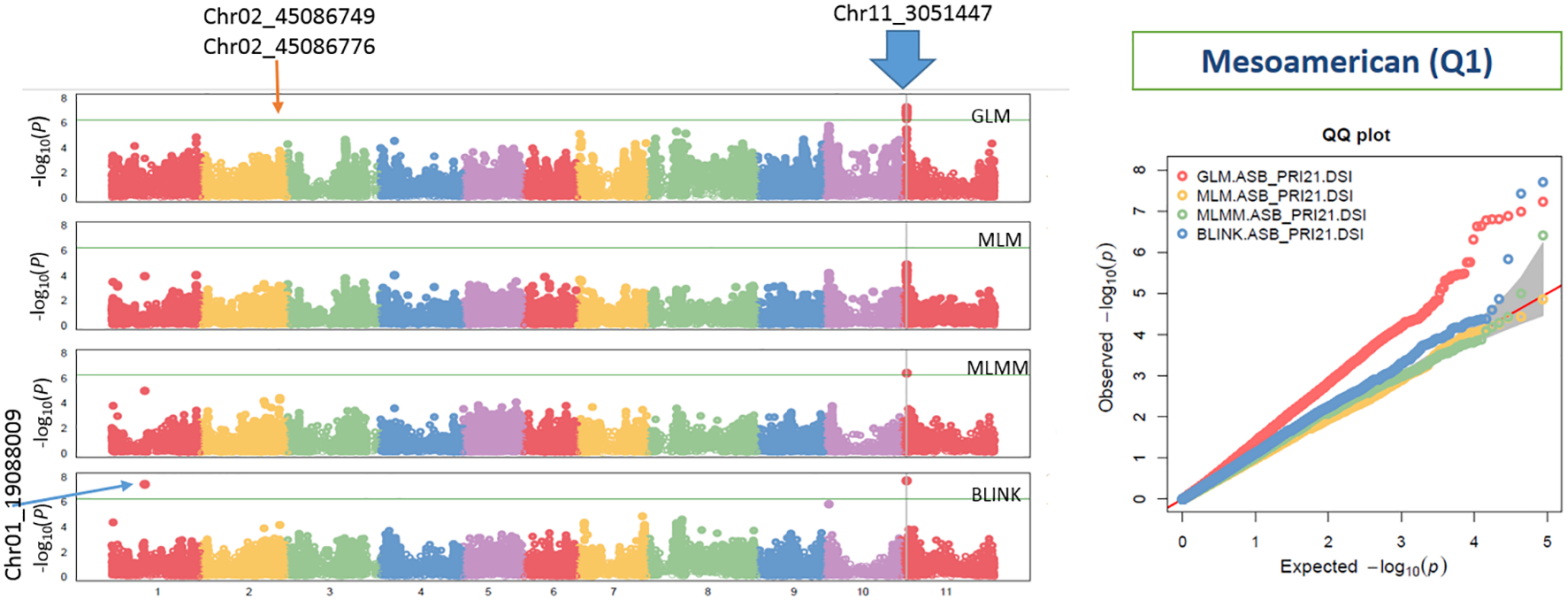
Distribution of Manhattan plots (left) and QQ plots (right) of GWAS for ashy stem blight isolate PRI21 disease severity index (DSI) on an association panel consisting of 194 accessions of Mesoamerican origin (Q1) based on GLM, MLM, MLMM, and BLINK in GAPIT3. For the Manhattan plot (left), the x-axis presents the common bean 11 chromosomes and the y-axis for LOD [−log(*p*-value)] value. For the QQ plot (right), the x-axis presents LOD [−log(*p*-value)] value and y-axis for expected LOD [−log(*p*-value)] value. GWAS, genome-wide association study; GLM, general linear model; MLM, mixed linear model; MLMM, multiple-locus MLM; BLINK, Bayesian-information and Linkage-disequilibrium Iteratively Nested Keyway; LOD, logarithm of odds.

**Figure 5:**
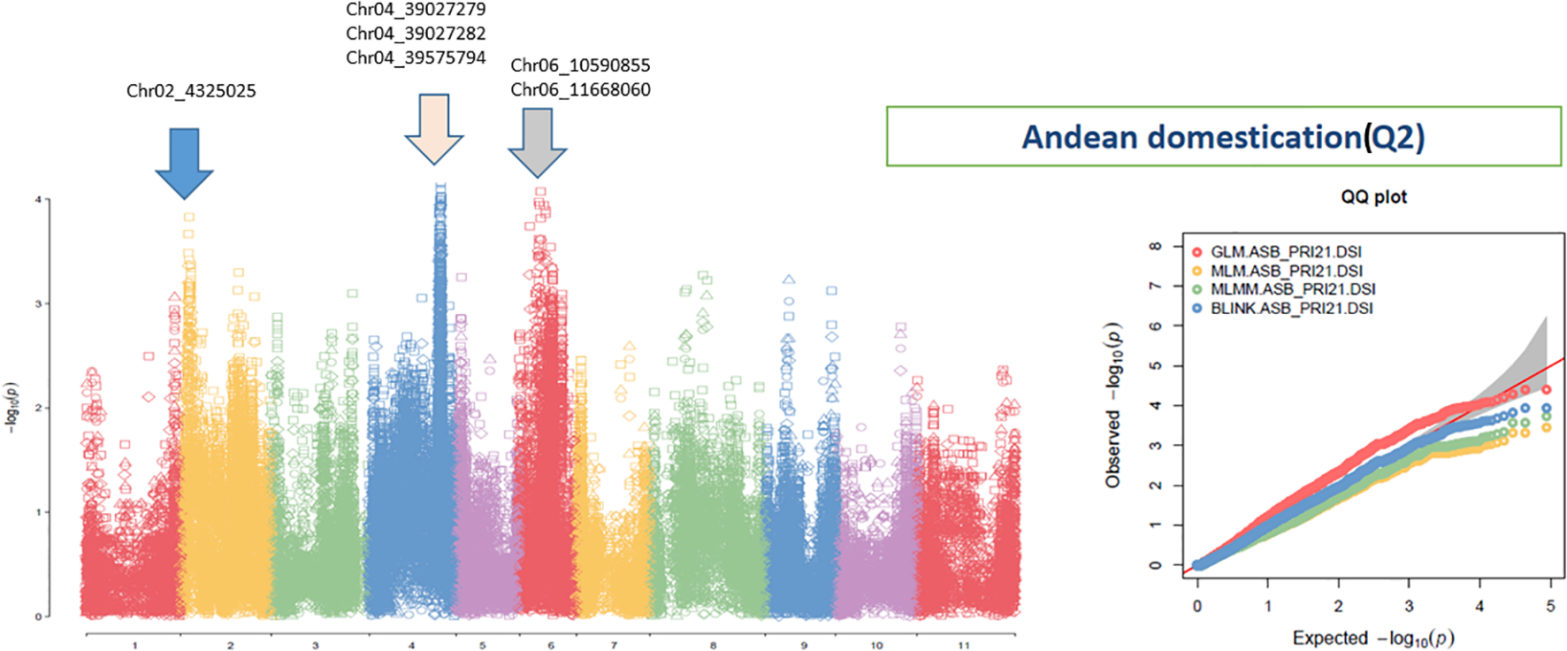
Distribution of Manhattan plot (left) and QQ plots (right) of GWAS for ashy stem blight isolate PRI21 disease severity index (DSI) on an association panel consisting of 141 accessions of Andean domestication (Q2) based on GLM, MLM, MLMM, and BLINK in GAPIT3. For the Manhattan plot (left), the x-axis presents the common bean 11 chromosomes and the y-axis for LOD [−log(*p*-value)] value. For the QQ plot (right), the x-axis presents LOD [−log(*p*-value)] value and y-axis for expected LOD [−log(*p*-value)] value. GWAS, genome-wide association study; GLM, general linear model; MLM, mixed linear model; MLMM, multiple-locus MLM; BLINK, Bayesian-information and Linkage-disequilibrium Iteratively Nested Keyway; LOD, logarithm of odds.

The analysis using the four models revealed multiple significant SNPs across the panels. The QQ plots (**Figures 3**–**5**) showed a significant deviation from the expected distribution in the all set (**Figure 3**, right half), Q1 (**Figure 4**, right), and Q2 (**Figure 5**, right), indicating the presence of SNPs associated with ASB resistance. Additionally, Manhattan plots (**Figures 3**-**5**), which cover all 87,193 tested SNPs, revealed several SNPs with LOD values greater than 6.2, 4.0, or 3.5, indicating SNPs associated with ASB resistance in each of the three panels.

To select significant SNP markers, a threshold of LOD > 6.25 was applied for all three panels, but due to the limited number of SNPs with such high LOD values, additional thresholds of LOD > 4.0 for the all set and Q1 panels and LOD > 3.5 for the Q2 panel were also used. A total of 64 SNPs were selected as significant across the panels (**Supplementary Table S3**).

**Figure 6:**
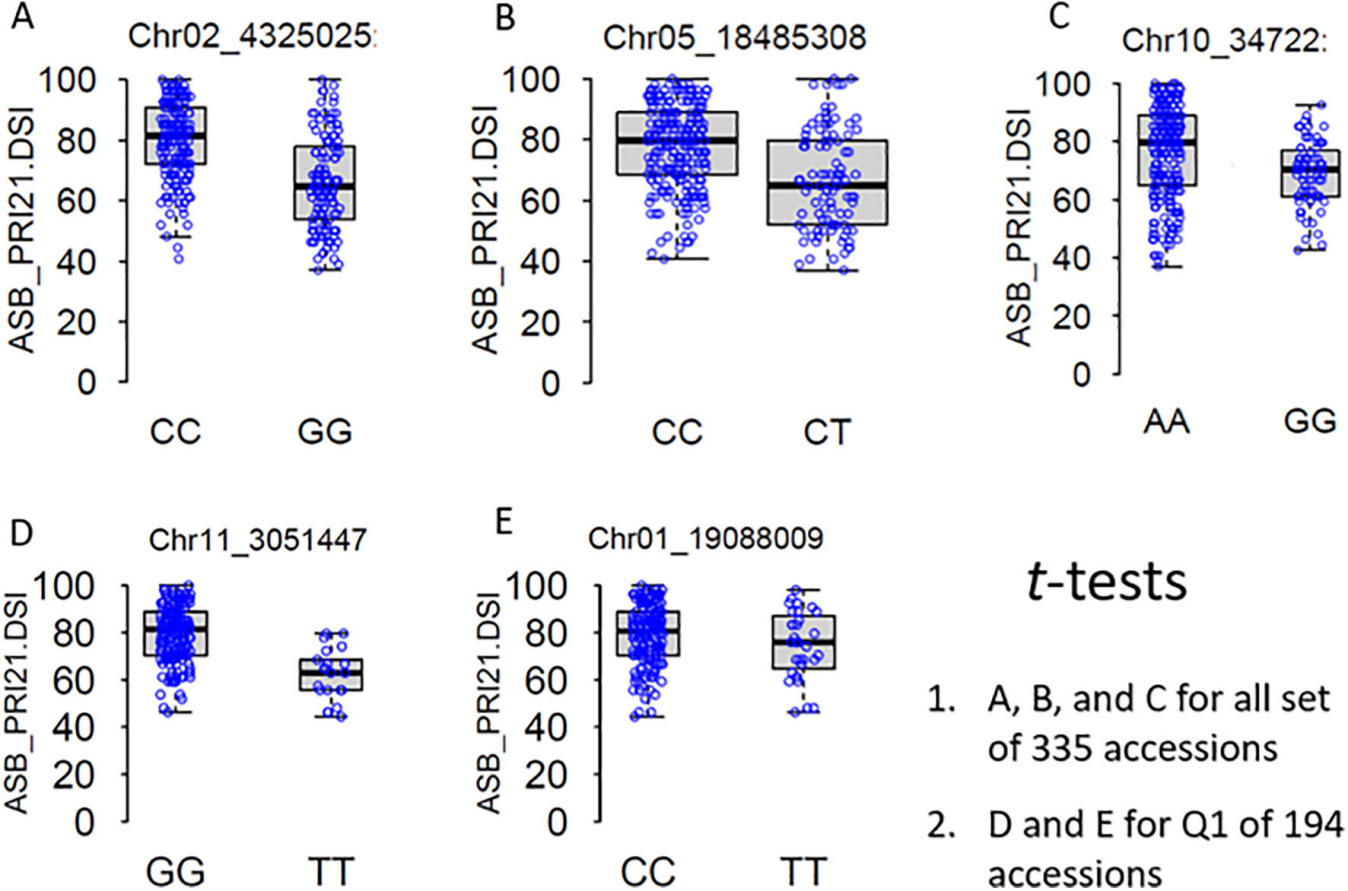
*t*-Tests of five significantly associated SNP markers for ashy stem blight isolate PRI21 disease severity index (DSI) on 335 USDA common bean accessions **(A–C)** and Q1 of 194 accessions **(D, E)**. SNP, single-nucleotide polymorphism.

Several SNP markers were identified as associated with ASB resistance in the different panels. In the all set, four SNPs were found to be significant: Chr02_4325025 (4,325,025 bp on chromosome 2), Chr04_39575794 (39,575,794 bp on chromosome 4), Chr05_18485308 (18,485,308 bp on chromosome 5), and Chr10_34722 (34,722 bp on chromosome 10). These SNPs exhibited LOD values greater than 6.25 in the BLINK model, with Chr02_4325025 and Chr10_34722 showing LOD values above 10 in the GLM and three of them also showing significance in the *t*-test (**Table 2**, **Figure 6**), suggesting the presence of QTLs in these regions.

**Table 2:** List of the 12 SNP markers associated with the resistance to *Macrophomina phaseolina* PRI21 isolate based on BLINK, MLMM, MLM, and GLM, and a *t*-test in three association panels (all, Q1, and Q2).

SNP	Chr	Pos	MAF %	LOD (−log(*p*))					Significant model (LOD > 6.25)	Resistance allele	Susceptible allele	Phenotype variance explained (PVE) (%)	Model for PVE	Set
				BLINK	MLMM	MLM	GLM	*t*-Test						
Chr02_4325025	2	4,325,025	40.3	7.42	5.01	4.77	10.68	16.76	Blink, GLM	G	C	5.76	BLINK	all
Chr04_39575794	4	39,575,794	10.4	8.63	4.22	4.06	4.22	0.78	Blink	G	A	7.45	BLINK	
Chr05_18485308	5	18,485,308	14.5	8.71	4.18	4.02	2.73	8.02	Blink	T	C	6.71	BLINK	
Chr10_34722	10	34,722	23.3	9.38	2.63	2.57	10.82	5.22	Blink, GLM	G	A	11.45	BLINK	
Chr01_19088009	1	19,088,009	17.5	7.42	5.00	3.95	3.16	1.24	Blink	T	C	12.31	BLINK	Q1
Chr02_45086749	2	45,086,749	22.4	4.17	4.28	3.04	3.23	6.81		A	G			
Chr02_45086776	2	45,086,776	22.6	4.17	4.42	3.13	3.29	6.81		C	T			
Chr11_3051447	11	3,051,447	11.3	7.70	6.41	4.86	7.23	10.51	Blink, MLMM, GLM	T	G	26.84	BLINK	
												43.70	MLMM	
Chr02_4325025	2	4,325,025	35.5	3.20	2.29	2.20	3.66	7.61		G	C			Q2
Chr04_39027279	4	39,027,279	39.7	3.94	3.58	3.32	4.41	0.96		A	G			
Chr04_39027282	4	39,027,282	39.7	3.94	3.58	3.32	4.41	0.96		G	A			
Chr04_39575794	4	39,575,794	24.8	3.48	3.04	2.87	3.94	2.99		G	A			
Chr06_10590855	6	10,590,855	0.16	3.50	3.01	2.84	3.97	5.78		G	T			
Chr06_11668060	6	11,668,060	0.16	3.61	3.09	2.91	4.08	5.78		G	T			

SNP, single-nucleotide polymorphism; BLINK, Bayesian-information and Linkage-disequilibrium Iteratively Nested Keyway; MLM, mixed linear model; GLM, general linear model; LOD, logarithm of odds; MAF, minor allele frequency.

In the Q1 panel, several SNPs were associated with ASB resistance, including Chr01_19088009 (19,088,009 bp on chromosome 1), Chr02_45086749 and Chr02_45086776 (45,086,749 bp and 45,086,776 bp, respectively, on chromosome 2), and Chr11_3051447 (3,051,447 bp on chromosome 11). SNP Chr01_19088009 had a high LOD value of 7.42 in the BLINK model and values above 3.0 in the other models. Both Chr02_45086749 and Chr02_45086776 on chromosome 2 had LOD values greater than 3.2 across all four models. Chr11_3051447 showed LOD values of 7.70, 6.41, 4.86, and 7.23 in BLINK, MLMM, MLM, and GLM, respectively, and a high LOD of 10.51 in the *t*-test (**Table 2**, **Figure 6**), indicating the presence of a QTL for ASB resistance on chromosome 11 in the Q1 panel (Mesoamerican origin).

In the Q2 panel, SNPs associated with ASB resistance included Chr02_4325025 (4,325,025 bp on chromosome 2); Chr04_39027279, Chr04_39027282, and Chr04_39575794 (39,027,279 bp, 39,027,282 bp, and 39,575,794 bp, respectively, on chromosome 4); and Chr06_10590855 and Chr06_11668060 (10,590,855 bp and 11,668,060 bp, respectively, on chromosome 6). These six SNPs exhibited LOD values ranging from 2.20 to 4.41 across the four GWAS models, suggesting a moderate association with ASB resistance in the Q2 panel.

### Candidate genes for ASB resistance

A total of 181 genes (**Supplementary Table S4**) were identified within 50-kb upstream or downstream of 64 SNP markers (**Supplementary Table S3**) associated with ASB disease scores and severity (DSI) using the common bean genome reference *P. vulgaris* 442_v2.1 from Phytozome. Among these, nine were identified as disease resistance gene analogs (**Table 3**).

**Table 3:** List of nine disease resistance gene analogs located at 50-kb distances upstream and downstream from seven out of 64 SNP markers in **Supplementary Table S3** and three genes closely linked within 3-kb distance from five out of 12 associated SNP markers for ashy stem blight resistance in **Table 2**.

Gene	Chr	Gene_start (bp)	Gene_end (bp)	Gene readable description	SNP	Chr	Pos (bp)	From.gene.Start (bp)	From.gene.Start (bP)	Distance	GWAS.set
*Phvul.002G046300*	2	4,279,849	4,285,111	Leucine-rich repeat (LRR) family protein	Chr02_4325025	2	4,325,025	45,176	39,914	<40 kb	All
*Phvul.002G046500*	2	4,293,694	4,296,160	Receptor-like protein kinase 1				31,331	28,865	<30 kb	
*Phvul.006G041400*	6	13,540,288	13,545,325	NB-ARC domain-containing disease resistance protein	Chr06_13527255	6	13,527,255	−13,033	−18,070	<15 kb	all.Q2
*Phvul.007G248100*	7	37,098,956	37,107,864	*S*-Adenosyl-L-methionine-dependent methyltransferase superfamily protein	Chr07_37155893	7	37,155,893	56,937	48,029	<50 kb	Q1
*Phvul.007G261400*	7	38,309,666	38,327,735	P-loop containing nucleoside triphosphate hydrolase superfamily protein	Chr07_38376380	7	38,376,380	66,714	48,645	<50 kb	Q1
*Phvul.007G261700*	7	38,373,507	38,374,058	Disease resistance-responsive (dirigent-like protein) family protein	Chr07_38376380	7	38,376,380	2,873	2,322	<3 kb	Q1
					Chr07_38407897	7	38,407,897	34,390	33,839	<35 kb	Q1
*Phvul.007G262000*	7	38,400,117	38,405,834	P-loop containing nucleoside triphosphate hydrolase superfamily protein	Chr07_38376380	7	38,376,380	−23,737	−29,454	<25 kb	Q1
					Chr07_38407897	7	38,407,897	7,780	2,063	<3 kb	Q1
*Phvul.010G018400*	10	2,689,239	2,693,034	Disease resistance protein (TIR-NBS-LRR class) family	Chr10_2726250	10	2,726,250	37,011	33,216	<35 kb	Q1
*Phvul.011G033000*	11	3,041,024	3,046,078	Leucine-rich repeat protein kinase family protein	Chr11_3051447	11	3,051,447	10,423	5,369	<5 kb	Q1
*Phvul.002G281800*	2	45,089,209	45,091,293	Homeodomain-like superfamily protein	Chr02_45086749	2	45,086,749	−2,460	−4,544	<3 kb	Q1
					Chr02_45086776	2	45,086,776	−2,433	−4,517	<3 kb	Q1
*Phvul.011G033100*	11	3,054,485	3,058,682	Glycosyl hydrolase family 10 protein	Chr11_3051447	11	3,051,447	−3,038	−7,235	<3.1 kb	Q1
*Phvul.004G112700*	4	39,012,396	39,030,784	NAD(P)-binding Rossmann-fold superfamily protein	Chr04_39027279	4	39,027,279	14,883	−3,505	On gene	Q2
					Chr04_39027282	4	39,027,282	14,886	−3,502	On gene	Q2

SNP, single-nucleotide polymorphism.

Notably, two genes, *Phvul.002G046300* [leucine-rich repeat (LRR) family protein] and *Phvul.002G046500* (receptor-like protein kinase 1), are located between 4,279,849 and 4,285,111 and 4,293,694 to 4,296,160 bp, respectively, on chromosome *Pv*02, separated by 8,532 bp. These two genes are positioned within 40 kb of the associated SNP marker Chr02_4325025, based on an analysis of 335 common bean accessions (**Table 3**). This suggests their potential involvement in ASB resistance. Another gene, *Phvul.006G041400* (NB-ARC domain-containing disease resistance protein), is located between 13,540,288 and 13,545,325 bp on chromosome *Pv*06, just 15 kb from the associated SNP Chr06_13527255. This association is consistent across both the Q1 and all-panel analyses, indicating that *Phvul.006G041400* may also contribute to ASB resistance. On chromosome *Pv*07, four genes were identified as potential ASB resistance candidates, based on their proximity to three associated SNP markers (Chr07_37155893, Chr07_38376380, and Chr07_38407897) within 50 kb in the Q1 panel (**Table 3**). These genes include the following:

- *Phvul.007G248100* (*S*-adenosyl-L-methionine-dependent methyltransferase superfamily protein), located from 37,098,956 to 37,107,864 bp;
- *Phvul.007G261400* (P-loop containing nucleoside triphosphate hydrolase superfamily protein), located from 38,309,666 to 38,327,735 bp;
- *Phvul.007G261700* [disease resistance-responsive (dirigent-like protein) family protein], located from 38,373,507 to 38,374,058 bp; and
- *Phvul.007G262000* (P-loop containing nucleoside triphosphate hydrolase superfamily protein), located from 38,400,117 to 38,405,834 bp.

Additionally, two more genes were identified:

- *Phvul.010G018400* [disease resistance protein (TIR-NBS-LRR class) family], located from 2,689,239 to 2,693,034 bp on chromosome *Pv*10, and
- *Phvul.011G033000* (leucine-rich repeat protein kinase family protein), located from 3,041,024 to 3,046,078 bp on chromosome *Pv*11, situated within 35 kb of SNP markers Chr10_2726250 and Chr11_3051447, respectively, in the Q1 panel (**Table 3**), suggesting that these genes may also be linked to ASB resistance.

In addition to these disease resistance gene analogs, three genes located within 5 kb of 12 associated SNP markers listed in **Table 2** were identified (**Table 3**). These include the following:

- *Phvul.002G281800* (homeodomain-like superfamily protein), located between 45,089,209 and 45,091,293 bp on chromosome 2, near SNP markers Chr02_45086749 and Chr02_45086776;
- *Phvul.011G033100* (glycosyl hydrolase family 10 protein), located from 3,054,485 to 3,058,682 bp on chromosome *Pv*11, near SNP marker Chr11_3051447; and
- *Phvul.004G112700* (NAD(P)-binding Rossmann-fold superfamily protein), located from 39,012,396 to 39,030,784 bp on chromosome *Pv*04, associated with two SNP markers Chr04_39027279 and Chr04_39027282, based on the Q2 panel.

These findings suggest that these genes are strong candidates for contributing to ASB resistance in common bean.

### Genetic diversity and utilization of the ASB-resistant germplasm accessions

**Figure 7:**
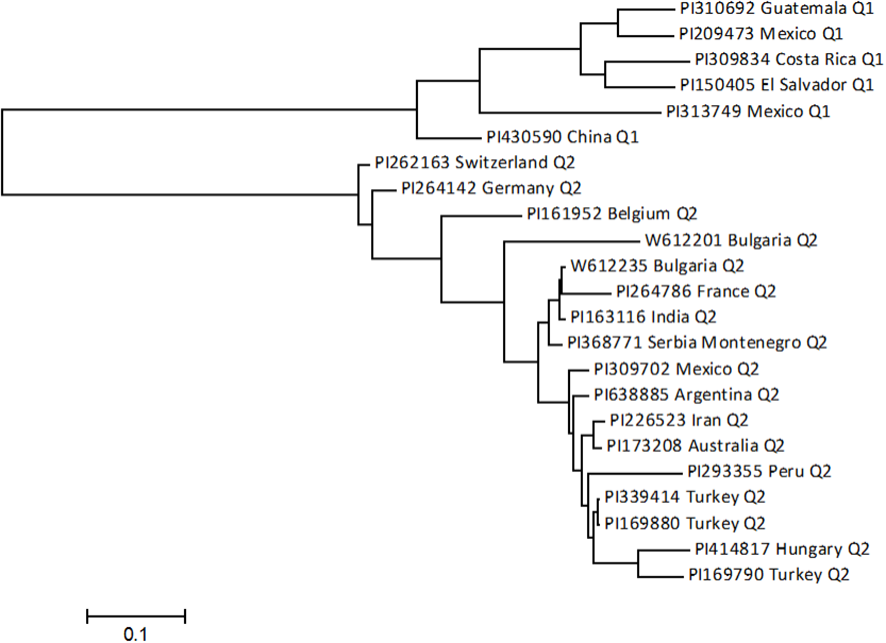
The phylogenetic tree was created using the maximum likelihood (ML) method from MEGA 7 in 23 intermediate-resistant common bean accessions.

Among the 23 accessions with intermediate resistance PRI21 *M. phaseolina* isolate (**Table 1**, **Figure 7**), six accessions belong to the Mesoamerican origin (Q1) and 17 to Andean domestication (Q2). Of the six accessions in Q1, two are from Mexico, and one each is from China, Costa Rica, El Salvador, and Guatemala. The 17 accessions in Q2 are sourced from 14 countries, with three accessions from Mexico, two from Bulgaria, and one from each of the other 12 countries (**Table 1**).

## Discussion

To the best of our knowledge, this is the first GWAS to evaluate ASB resistance across the USDA GRIN collection from widely geographic regions. In fact, 62 SNPs were identified to be associated with resistance to *M. phaseolina* isolate PRI21 across three common bean panels: all (335 accessions), Mesoamerican group Q1 (194 accessions), and Andean group Q2 (141 accessions). The Chr01_19088009 SNP on *Pv*01; Chr02_4325025, Chr02_45086749, and Chr02_45086776 on *Pv*02; and Chr11_3051447 SNP on *Pv*11 chromosomes are novel QTLs identified to confer resistance to ASB in this research. Previous studies has identified resistant QTLs on chromosomes *Pv*03, *Pv*04, *Pv*05, *Pv*06, *Pv*07, *Pv*08, *Pv*09, and *Pv*10 (Méndez et al., 2017; Miklas et al., 1998b; Viteri et al., 2022, 2024b). Furthermore, the QTL on the *Pv*11 chromosome is the first derived from the Middle American gene pool that explained >25% of phenotypic variance to ASB. This is an important discovery because Middle American beans have lower levels of resistance compared with Andean common beans (Viteri and Linares, 2017, 2022a). In fact, the QTL on chromosome *Pv*01 also derived from the Middle American gene pool explained 12% of the phenotypic variance as other minor QTLs from Middle American common beans BAT 477 and XAN 176 that reached values below 20% (Miklas et al., 1998b; Viteri et al., 2022). Conversely, a major QTL on the *Pv*07 chromosome identified in Andean genotype PRA154 explained close to 40% of the phenotypic variance to PRI21 *M. phaseolina* isolate (Viteri et al., 2024a).

*Phvul.003G175900* (drought sensitive, WD repeat-containing protein 76) on chromosome *Pv*03 and derived from BAT 477 (Viteri et al., 2022) and *Phvul.007G173900* [methylcrotonyl-CoA carboxylase alpha chain, mitochondrial 3-methylcrotonyl-CoA carboxylase 1 (MCCA)] located on chromosome *Pv*07 and derived from PRA154, respectively, were the only candidate genes reported for ASB resistance so far (Viteri et al., 2022, 2024a). Thus, these genes and those identified in this study [e.g., *Phvul.002G046300* (LRR), *Phvul.002G046500* (receptor-like protein kinase 1), and *Phvul.004G112700* (NAD(P)-binding Rossmann-fold superfamily protein)] and *Phvul.011G033100* gene (glycosyl hydrolase family 10), among others located on *Pv*02, *Pv*04, *Pv*05, *Pv*6, *Pv*10, and *Pv*11 chromosomes and referred in **Table 3**, may be used for MAS to improve the levels of resistance to ASB. For instance, it has been reported that the leucine-rich repeat receptor-like kinase (LRR-RLK) genes are associated with resistance to *M. phaseolina* in *Sesame indicum* L. (Yan et al., 2024), white mold [caused by the necrotrophic fungus *Sclerotinia sclerotiorum* (Lib.) de Bary] in snap bean (Arkwazee et al., 2022), and powdery mildew (caused by *Erysiphe polygoni* DC) in common bean (Binagwa et al., 2021). Also, the RLK genes were reported to be associated with plant resistance response against pathogens in various legumes including common bean (Restrepo-Montoya et al., 2021). Furthermore, the glycosyl hydrolase genes were reported to be associated with mechanisms of defense against hemibiotrophic or necrotrophic fungi in *Arabidopsis thaliana* (L.) Heynh (Kim et al., 2022). Thus, future studies should be focused on verifying which of these genes/QTLs or others had a major effect on resistance to ASB in common bean populations with different genetic backgrounds. For this, the development of recombinant inbred lines (RILs) between PI 161952, PI 163116, PI 173208, PI 262163, and PI 430590 that showed higher levels of resistance (scores between 3.3 and 4.0) crossed with highly susceptible common bean cultivars from different market classes (e.g., pinto ‘Othello’, white ‘Verano’, or black ‘Zorro’; Viteri and Linares, 2017, 2022a; Viteri et al., 2019) should be recommended. These RILs may be screened against *M. phaseolina* isolates with different levels of virulence and at different growth stages as in previous studies (Vázquez et al., 2024; Viteri et al., 2022). Also, genotyping with the SNPs reported in this study or other suitable molecular markers linked with the mentioned genes or others would be necessary for the QTL analysis.

Although there is no complete resistance (scores ≤ 3) in the majority of 335 genotypes in this study, the PI 173208 and PI 264786 accessions had the lower ASB scores (3.5 and 3.3, respectively). Both genotypes are under the Andean gene pool, supporting previous studies where Andean genotypes (e.g., A 195, PRA154, and PRA155) had higher levels of resistance to ASB (Viteri and Linares, 2017, 2022; 2022a, Viteri et al., 2024a). The PI 173208 and PI 264786 common bean accessions can be crossed with UPR-Mp breeding lines or common bean genotypes (e.g., A 195, BAT 477, ‘Badillo’, ‘PC 50’, PRA154, PRA155, VA 19, and XAN 176), which have intermediate or complete resistance to *M. phaseolina* in different environments (Mayek et al., 2001b; Miklas et al., 1998b; Viteri et al., 2019, 2023, 2024a; Viteri and Linares, 2017, 2022a). Furthermore, crosses with the scarlet runner bean accession PI 183412 that showed resistance scores (<3.5) (Viteri and Miranda, 2024) can be conducted. Backcrossing, gamete selection, and/or recurrent selection breeding methods should be employed to incorporate higher levels of resistance to *M. phaseolina* isolates between two- and multiple-parent populations including *P. vulgaris* and/or *P. coccineus* genotypes (Schwartz et al., 2006; Terán and Singh, 2010; Viteri et al., 2023). It is strongly recommended to conduct genetic studies and breeding for resistance to ASB in the greenhouse for the following reasons: 1) a direct exposition with the pathogen is necessary to avoid disease escapes, 2) multiple inoculations with *M. phaseolina* isolates with different levels of virulence can be carried out to select genotypes with specific of broad-spectrum ASB resistance, 3) less expenses are incurred in labor and field activities to select resistant or partially resistant genotypes, 4) the correct identification of true physiological resistance is guaranteed, 5) an appropriated environment for the disease development is promoted, and 6) the co-infection with other pathogens that may affect the ASB severity is avoided. The efficacy of screening in the greenhouse against soil-borne fungi such as *M. phaseolina* and *S. sclerotiorum* has been well documented (Singh et al., 2014; Terán and Singh, 2009; Viteri et al., 2015, 2017, 2022a; Viteri and Miranda, 2024).

## Conclusion

This study aimed to identify common bean accessions with resistance to *M. phaseolina* isolate PRI21, the causal agent of ashy stem blight. Using four different GWAS models (GLM, MLM, MLMM, and BLINK) and 87,193 SNPs from whole-genome resequencing, the analysis revealed 62 SNPs associated with ASB resistance across three panels: all accessions (335), Mesoamerican origin (Q1, 194 accessions), and Andean origin (Q2, 141 accessions). Key SNPs were identified on chromosomes *Pv*01, *Pv*02, *Pv*04, *Pv*05, *Pv*06, *Pv*10, and *Pv*11, with several SNPs exhibiting high LOD values, indicating strong associations with ASB resistance. Among the 23 accessions with intermediate resistance, six were of Mesoamerican origin, and 17 were of Andean origin. These accessions, along with their identified SNP markers, are potential resources for breeding programs aimed at improving ASB resistance. The study also highlights the importance of marker-assisted selection and the use of conventional breeding techniques to improve the levels of ASB resistance in the development of future common bean germplasm/cultivars.

## Data Availability

The datasets presented in this study are available in the **Supplementary Materials**. The SNP data can be accessed on FigShare at https://doi.org/10.6084/m9.figshare.28464020.v1. The accession numbers used in this study are provided in the article and **Supplementary Materials** for reference.
